# Selective Vapor Permeation Behavior of Crosslinked PAMPS Membranes

**DOI:** 10.3390/polym12040987

**Published:** 2020-04-24

**Authors:** Ye Ji Son, So Jeong Kim, Young-Jin Kim, Kyung-Hye Jung

**Affiliations:** 1 School of Advanced Materials and Chemical Engineering, Daegu Catholic University, Gyeongsan 38430, Korea; dpwl1031@naver.com (Y.J.S.); sojyngdldy@naver.com (S.J.K.); 2 Department of Biomedical Engineering, Daegu Catholic University, Gyeongsan 38430, Korea; yjkim@cu.ac.kr

**Keywords:** polymer membranes, selective permeation, poly(2-acrylamido-2-methyl-1-propanesulfonic acid), crosslinking, chemical protection, nanofibers

## Abstract

The effect of crosslinking on vapor permeation behavior of polyelectrolyte membranes was studied. Poly(2-acrylamido-2-methyl-1-propanesulfonic acid) (PAMPS) membranes were crosslinked by using crosslinkers with different lengths between the reactive ends. Crosslinked membranes with a longer crosslinking length showed lower water vapor permeability due to the lower sorption coefficient. It was also shown that the permeation behavior of PAMPS membranes was more affected by sorption than diffusion. For chemical protection applications, the ratio of water over chemical warfare agent permeability (i.e., selectivity) was measured. Due to the high water solubility of polyelectrolytes, crosslinked PAMPS allowed for the selective permeation of water over harmful chemical vapor, showing a selectivity of 20. The addition of electrospun nylon nanofibers in the membranes significantly improved the selectivity to 80, since the embedded nanofibers effectively reduced both diffusion and sorption coefficients of chemical warfare agents.

## 1. Introduction

One of the key properties of polymer membranes is to control the permeation behavior of various chemical species through the membrane. Selective permeable membranes especially have a broad range of applications, such as filtration, gas separation, medical applications [1,2,3,4].

Permeation occurs through three steps: sorption (adsorption and dissolution), diffusion, and desorption. Permeation behavior is mainly determined by the sorption and diffusion of permeants in the membrane matrix. For nonporous membranes, the interactions between permeants and membrane materials dominate sorption behavior while the size of permeants and the structure of the membrane matrix dominate diffusion behavior. The selective permeation between two permeants is also determined by the sorption and diffusion selectivity. For polymer membranes, their free volume and functional groups can be the critical factors to control selective permeation behaviors. Therefore, many studies focus on the modification of molecular structure of the polymer matrix to obtain ideal permeability [5]. 

Crosslinking is one of the most typical and effective ways to modify the molecular structures and performance of polymers. It has been widely known that selective permeability of gas separation membranes is improved by crosslinking polymers [6]. Optimizing mechanical properties of polymer membranes by crosslinking has also been reported [7,8,9,10]. In addition to crosslinking, incorporating nanofillers in the membranes is commonly studied to control membrane properties and performances [11,12,13,14]. 

One of the important applications of selective permeable membranes is chemical protection [15,16,17]. Effective protection against exposure to toxic and hazardous chemicals is the main purpose of using chemical protective membranes. At the same time, water vapor transport is also critical to secure breathability of users. Therefore, a high selectivity, which is the ratio of water over chemical wafer agents, of chemical protective membranes is mandatory. 

Poly(2-acrylamido-2-methyl-1-propanesulfonic acid) (PAMPS) is one of polyelectrolytes that shows reasonable water permeability due to the presence of hydrophilic sulfonic acid (–SO_3_H) groups. The hydrophilic nature of sulfonic groups promotes water sorption, contrasting the sorption of organic chemicals, which is desired for chemical protective membranes [18]. However, the high water solubility and low mechanical properties of PAMPS limit its wide applications, and these have been solved by blending polymers and crosslinking [19,20,21,22]. 

Nanofibers are ultrathin fiber mats that are generally fabricated by electrospinning. The large surface area to volume ratio and the interconnected porosity of nanofibers make them a good candidate for chemical protection applications by allowing moisture to penetrate through and by blocking chemical vapors [23]. In addition, composite nanofibers containing catalysts that are able to detoxify harmful compounds have been reported [24,25,26]. Nanofibers are also a good candidate for nanofillers of composite membranes since their large surface area and porosity can control selective permeations and mechanical properties. 

In this study, crosslinked PAMPS membranes were prepared with crosslinkers with a different chain length to study the effect of crosslinking on permeability. Sorption and diffusion were investigated to understand permeation behavior. Nanofiber-embedded PAMPS membranes were also prepared to control permeability, and their selectivity was studied for chemical protection applications. 

## 2. Experimental Section

We used 2,2′-azobis(2-methylpropionitrile) (AIBN; Junsei Chemical, Tokyo, Japan), 2-acrylamido-2-methyl-1-propanesulfonic acid (AMPS; Sigma-Aldrich, Tokyo, Japan), and dimethylsulfoxide (DMSO; TCI Chemicals, Tokyo, Japan) as an initiator, monomer, and solvent, respectively. Ethylene glycol diacrylate (EGDM), tetra(ethylene glycol) diacrylate (TEGDM), and poly(ethylene glycol) diacrylate (PEGDM) were purchased from Sigma-Aldrich and used as crosslinkers. Dimethyl methylphophonate (DMMP) used as a simulant for Sarin was purchased from Sigma-Aldrich.

**Figure 1:**
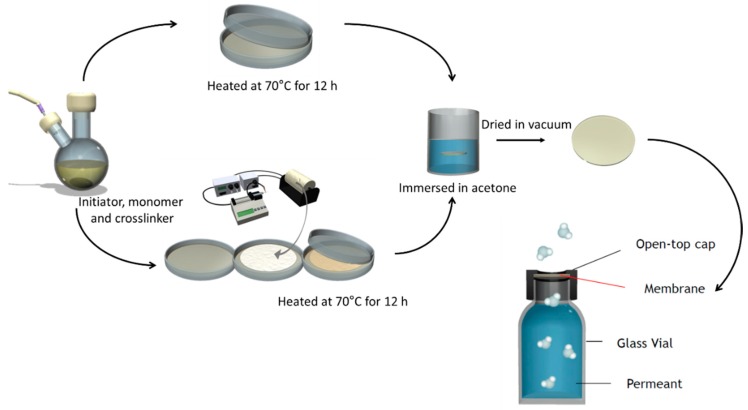
Scheme of membrane preparation and vapor permeation testing cell.

**Figure 2:**
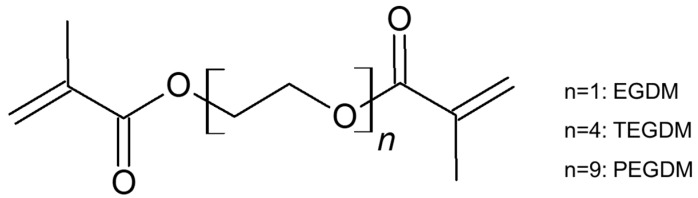
Structure of crosslinkers.

Figure 1 shows the scheme of membrane preparation. PAMPS membranes were synthesized by free radical polymerization. Here, 0.3 mol/L AIBN and 3 mol/L AMPS were dissolved in DMSO. Three crosslinkers, EGDM, TEGDM, and PEGDM (as shown in Figure 2), were also added in the mixtures, and the nominal crosslinking degree, which is the molar ratio of the feed crosslinkers to the monomer, is fixed at 10%. The mixtures were placed between two plates and heated at 70 °C for 12 h. Afterwards, they were soaked in acetone to remove unreacted components and dried in vacuum for 24 h.

Cross-sectional images of membranes were observed using a scanning electron microscope (SEM, SU8220, Hitachi, Tokyo, Japan). Attenuated total reflectance Fourier transform infrared (ATR-FTIR) spectra were obtained using a 4100, Jasco, Easton, MD, USA. to observe chemical composition of crosslinked PAMPS membranes. Mechanical properties of membranes were observed by tensile testing using a TO-101 universal testing machine (Testone Co., Siheung, South Korea) with a 2 kN load capacity at a rate of 10 mm/min. All membranes were cut into rectangular specimens with a size of 10 mm × 30 mm, and five parallel measurements were tested. The glass transition temperatures (*Tg*) of the synthesized membranes were obtained by differential scanning calorimetry (DSC) measurements using TA Instruments (Q2000, TA Instruments LLC, New Castle, DE, USA).

The ion exchange capacity (IEC, meq g^−1^) of the membranes was determined by the acid-base titration method. After soaking each membrane in a 2 M NaCl solution for 24 h to convert the protons to sodium ions, the remaining solution was titrated with an NaOH solution using phenolphthalein as an indicator. The IEC values of the membranes were calculated using Equation (1) as follows:

(1) $$\mathrm{IEC}=\frac{V_{\mathrm{NaOH}}\cdot C_{\mathrm{NaOH}}}{W_{\mathrm{dry}}}$$

where *V*_NaOH_ is the consumed volume of NaOH (mL), *C*_NaOH_ is the concentration of NaOH (M), and *W*_dry_ is the weight of dry membrane (g).

Nanofiber-embedded PAMPS membranes were also fabricated to see the effect of nanofillers. Nylon nanofibers were prepared by the electrospinning of a 17 wt % nylon solution in formic acid with an applied voltage of 18 kV. The surface of nylon nonwovens was observed using an SEM (JEOL’s JSM 6335F, Tokyo, Japan). Electrospun nylon nanofibers were soaked in the mixtures of AIBN, AMPS, TEGDM, and DMSO, and then heated for membrane fabrication, which is denoted as T10N.

The vapor permeation of membranes was evaluated using the modified method based on the American Society for Testing and Materials (ASTM) E96-95 procedure. Synthesized membranes were placed in open-top caps and glass vials filled with permeants, water, or DMMP, as shown in Figure 1. Theses permeation test cells were placed in a humidity/temperature environment chamber (35 °C and 50% RH, JEIO Tech, TH-PE-025, Seoul, Korea), and their weights were recorded every 24 h until the weight losses became constant. Vaper permeability (*P*, mol/m/s/mmHg) was calculated according to Equation (2) as follows:

(2) $$P=\frac{\Delta G\cdot l}{t\cdot A\cdot \Delta P}$$

where *ΔG* is the weight loss, *l* the membrane thickness, *t* the time, *A* the membrane area exposed to vapor, and *ΔP* the pressure differential across the membranes. Sorption coefficient (*S*, mol/m^3^/mmHg) was calculated using Equation (3) as follows:

(3) $$S=\frac{G_{s}}{V\cdot \Delta P}$$

where *G_s_* is the weight of the permeant absorbed in membranes, and *V* is the volume of the membranes. The diffusion coefficient (*D*, m^2^/s) was obtained based on permeability and the sorption coefficient using Equation (4) as follows:

(4) $$D=\frac{P}{S}$$

Selectivity (α) is the ratio of water permeability over DMMP permeability, and calculated using Equation (5) as follows:

(5) $$\alpha =\frac{P_{\mathrm{water}}}{P_{\mathrm{DMMP}}}$$

For each membrane, the measurements on at least five specimens with a thickness range of 0.50 ± 0.05 mm were conducted. 

## 3. Results and Discussion

The effect of crosslinking on membrane properties was studied by using three crosslinkers, EGDM, TEGDM, and PEGDM, with a crosslinking degree of 10%, which are denoted as E10, T10, and P10, respectively.

**Figure 3:**
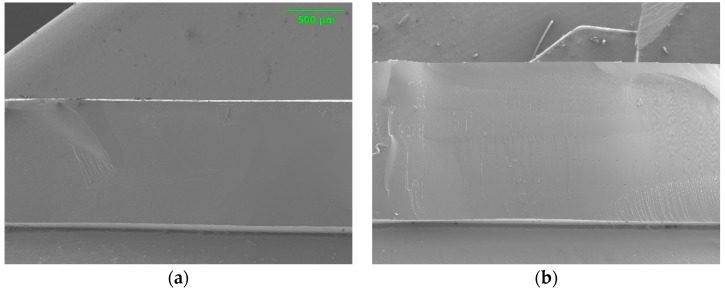

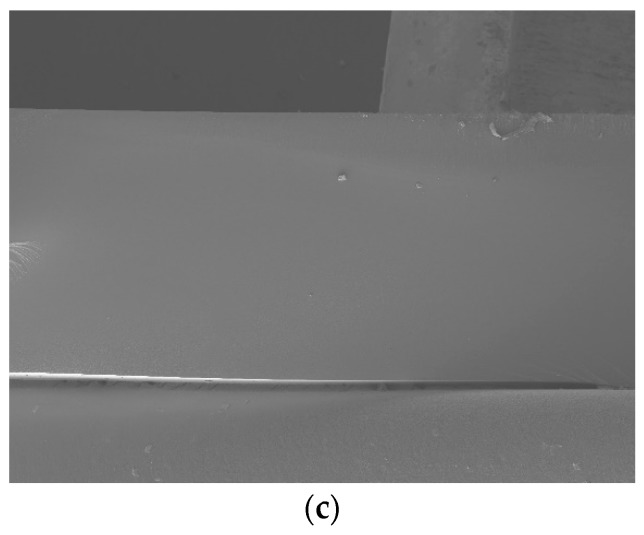
Cross-sectional SEM images of E10 (**a**), T10 (**b**), and P10 (**c**).

Figure 3 shows cross-sectional images of PAMPS membranes. Uniform morphology and microstructure along the cross section are observed without any void or defect for all three crosslinked PAMP membranes.

**Figure 4:**
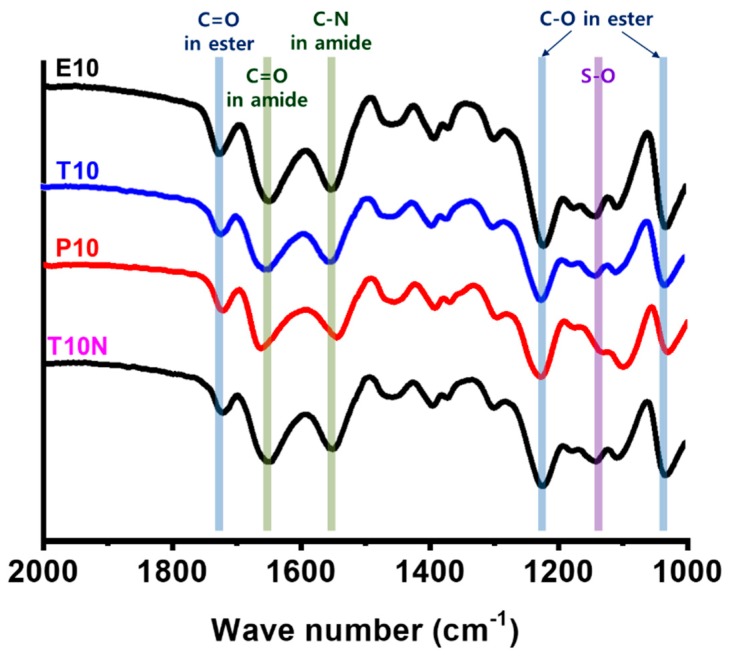
FTIR spectra of poly(2-acrylamido-2-methyl-1-propanesulfonic acid) (PAMPS) membranes.

Chemical composition of synthesized PAMPS membranes was investigated by ATR-FTIR spectroscopy, as shown in Figure 4. All four membranes show distinctive bands at 1635, 1550, and 1130 cm^−1^ for C=O, C–N, and S–O stretches, respectively. It is also seen that they exhibit peaks at 1750 cm^−1^ for C=O, and 1250 and 1050 cm^−1^ for C–O stretches of esters in crosslinkers. No significant changes with different crosslinkers can be found. 

In Table 1, tensile properties of PAMPS membranes were measured to see the effect of crosslinkers on mechanical properties. Young’s modulus, tensile strength, and elongation at break were calculated from the stress–strain curves. It is clearly seen that tensile properties are enhanced as the crosslinking length decreases, which confirms that crosslinking can effectively modify the mechanical properties of polymer membranes. 

**Figure 5:**
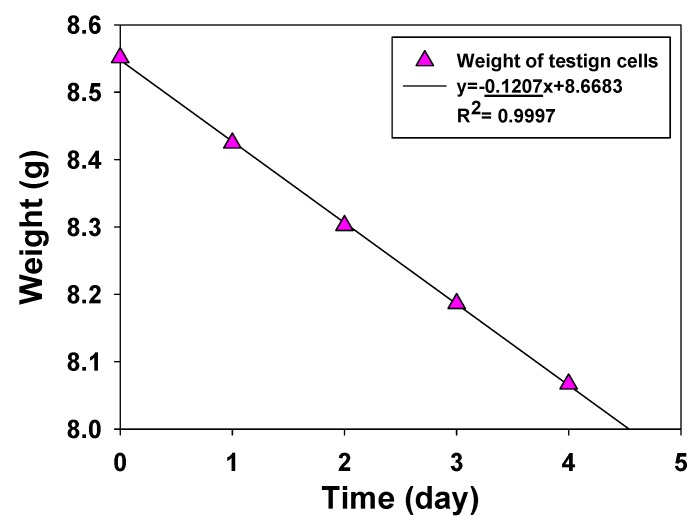
Typical water vapor transfer rate data of a PAMPS membrane.

Figure 5 shows the typical weight–time curve of the permeation testing cell, which represents the weight loss of water vapor penetrating membranes. For chemical protection applications, the water vapor transport rate is required to be greater than 2000 g/m^2^/day [27]. All PAMPS membranes fabricated in this study exhibit a water vapor transport rate higher than 2000 g/m^2^/day.

Crosslinking forms a chemical network by connecting linear polymer chains via the form of covalent bonds or ionic bonds and is frequently used to modify polymer structures and properties. Thus, the crosslinking of PAMPS is introduced to control its structures and permeation behavior. In addition, a high solubility of PAMPS in water can be prevented by crosslinking. 

**Figure 6:**
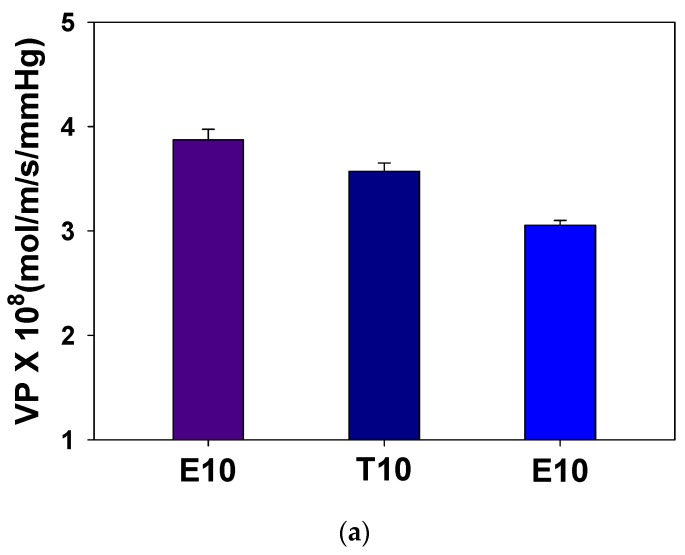

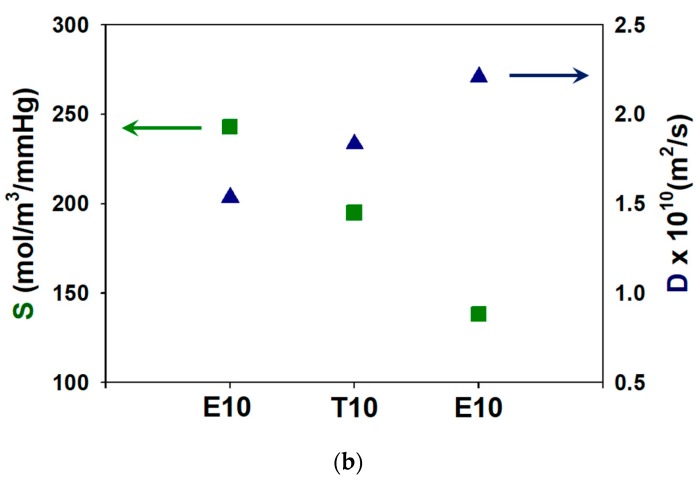
Effect of crosslinkers on (**a**) water permeabilities and (**b**) sorption and diffusion coefficients of crosslinked PAMPS.

In Figure 6a, it is seen that the permeability of crosslinked PAMPS is reduced as the crosslinker length increases. To understand the permeation behavior, the sorption and diffusion coefficients were also measured, as shown in Figure 6b. Sorption coefficients, which are the concentrations of vapors in the matrix at the equilibrium, decrease with an increase of the crosslinker length while diffusion coefficients increase. 

**Figure 7:**
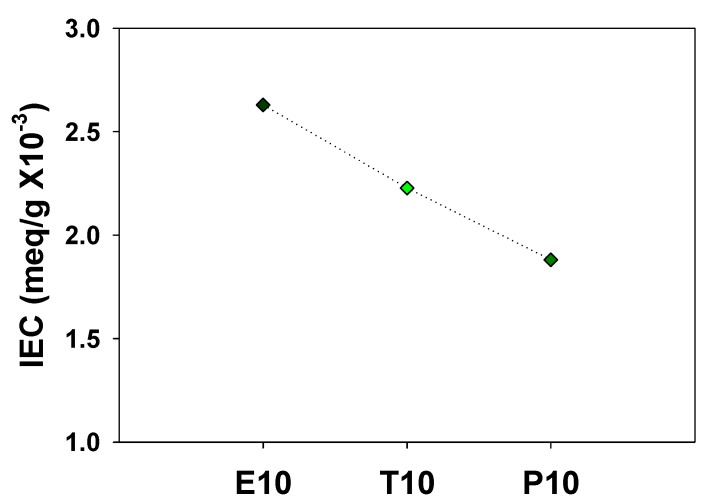
Ion exchange capacity (IEC) of PAMPS membranes.

High water permeability of polyelectrolytes is caused by the presence of hydrophilic ionic groups, such as the sulfonic acid for PAMPS. Crosslinked PAMPS membranes with a long crosslinker (P10) have a smaller number of sulfonic acid groups per unit mass compared to those with shorter ones, which results in the lower water sorption. In order to confirm this, the IEC was measured and shown in Figure 7. As expected, P10 contains the smallest number of sulfonic acid groups among these, resulting in the lowest water sorption. Sundarrajan et al. also reported that microporous polymers crosslinked with a shorter crosslinker exhibit higher gas adsorption capacity [24]. 

**Figure 8:**
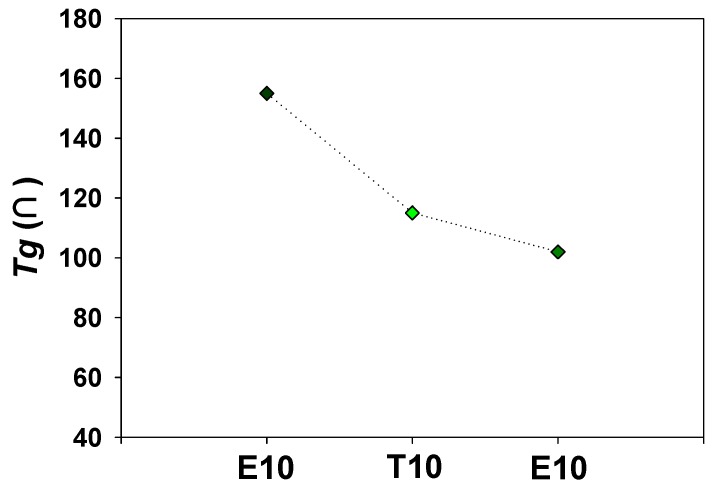
Effect of the crosslinker on glass transition temperatures of crosslinked PAMPS.

Figure 6b also shows that the diffusion coefficients increase with an increase in crosslinker lengths. The diffusion behavior of membranes is determined by the polymer chain flexibility, and thus *Tg* of E10, T10, and P10 was measured by DSC as shown in Figure 8. It can be clearly seen that the *Tg* is decreased as the crosslinking length increases. PAMPS crosslinked with longer crosslinkers exhibits high free volume and high flexibility of polymer chains, which promotes the vapor diffusion across the membrane matrix. PAMPS has a large number of sulfonic acid groups, which can form physical crosslinking between polymer chains. Chemical crosslinking disturbs the interactions between ionic species, and therefore, it improves polymer chain flexibility [28,29]. It was reported that crosslinked PAMPS membranes exhibit higher permeability than linear ones [30]. A long crosslinker chain may more effectively prevent these strong interactions, resulting in lower *Tg*. However, P10 exhibits the lowest water permeability despite the high polymer flexibility. It can be concluded that the water permeation of crosslinked PAMPS membranes is more affected by sorption than diffusion. 

Crosslinking has been used to control mechanical, chemical, and thermal stability by tailoring polymer chain flexibility and crystallinity [31]. It was reported that crosslinking polyelectrolytes increases their vapor permeability due to the improved polymer chain flexibility [30]. Moreover, PAMPS exhibits a lower permeability than other polyelectrolytes since hydrogen bonding between amide groups enhances the intermolecular interaction of polymer chains [32]. By contrast, crosslinking can decrease plasticization of the polymer matrix, causing a reduction in the permeability of polymer membranes such as cellulose acetate, polymers of intrinsic microporosity (PIM), and polyimides [33,34,35]. 

In addition to permeation behavior, crosslinking is also a useful method to modify the mechanical stability of polymer membranes. The effect of crosslinker lengths on the mechanical properties has been studied: Salimi-Kenari et al. reported that hydrogels crosslinked with a shorter crosslinker show a lower swelling ratio [36]. Caycik et al. reported that bend strength and tensile strength decreases as the chain lengths of crosslinkers increased while the impact strength increases [37]. 

Controlling permeation behavior is essential for many applications, including gas separation, filtration, and chemical protection. Among them, the ability to control the permeation of different species, i.e., selective permeability, is strongly required for chemical protective membranes. The most common means of exposure to chemical warfare agents is by vapor. For the protection of users, the membranes should block the permeation of toxic and hazardous chemical vapors. However, the permeation to water vapor or respiratory gases should be allowed for to avoid discomfort, heat stress, and fatigue of users. To obtain high selectivity of water over hazardous chemical vapors, the incorporation of nonwoven nanofibers as nanofillers is investigated.

**Figure 9:**
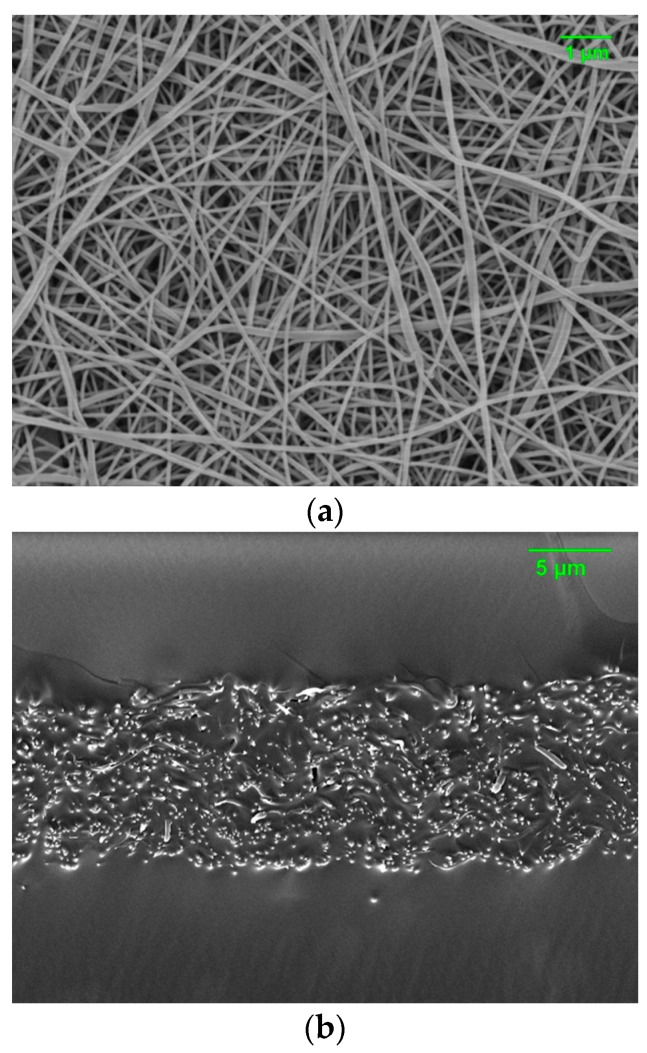
Surface morphology of electrospun nylon nanofibers (**a**) and cross-sectional image of T10N (**b**).

Nylon nanofibers were fabricated by the electrospinning method and inserted into T10 as a nanofiller, which is denoted as T10N. TEGDM was chosen as a crosslinker for the composite membranes since T10 shows moderate permeability, which can meet the requirements for chemical protection such as high water permeability and low organic chemical permeation. The content of the nylon nanofibers in the resultant composite membranes is about 10 wt %. Figure 9 displays electrospun nylon nanofibers with a fiber diameter of 98 nm and the cross-sectional image of T10N. It is shown that nylon nanofibers are well embedded inside of T10. As shown in Table 1, this can successfully improve the tensile strength and Young’s modulus of PAMPS membranes.

**Figure 10:**
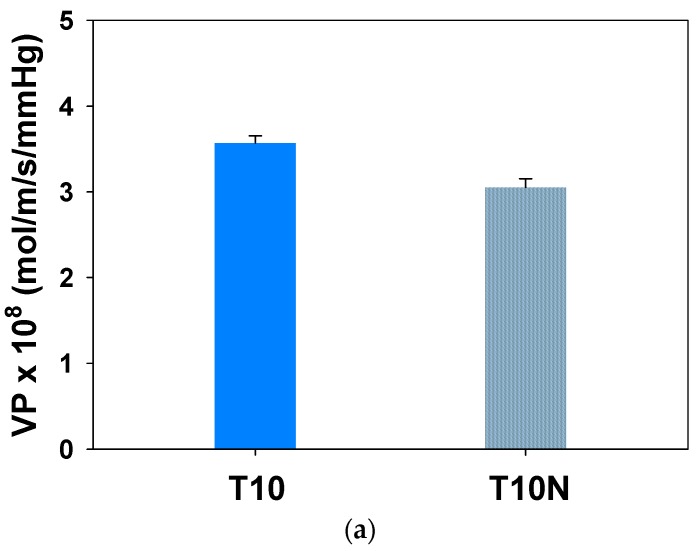

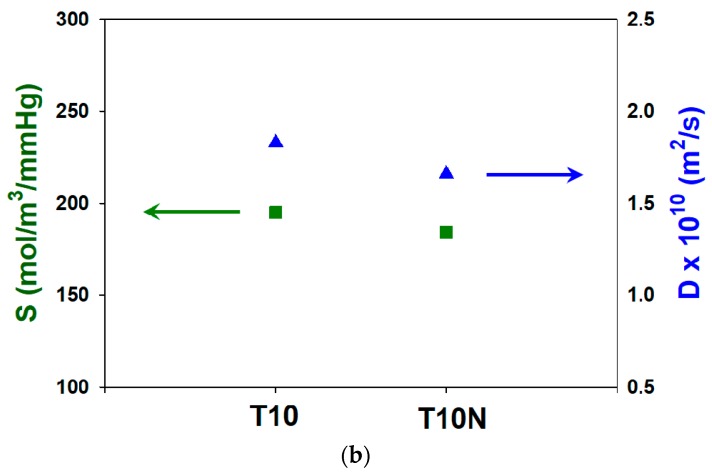
Water (**a**) permeabilities and (**b**) sorption and diffusion coefficients of crosslinked PAMPS.

Water vapor permeabilities of T10 and T10N membranes are shown in Figure 10a. Introducing nanofibers in PAMPS decreases water permeability by 14.6%. As shown in Figure 10b, a decrease in both sorption and diffusion leads to the lower permeability of T10N. The high hydrophilic nature of polyelectrolytes caused by ionic groups promotes the sorption and diffusion of water vapors. Compared to PAMPS, nylon is more hydrophobic, and thus, the presence of nylon nanofibers in PAMPS reduces water sorption.

**Figure 11:**
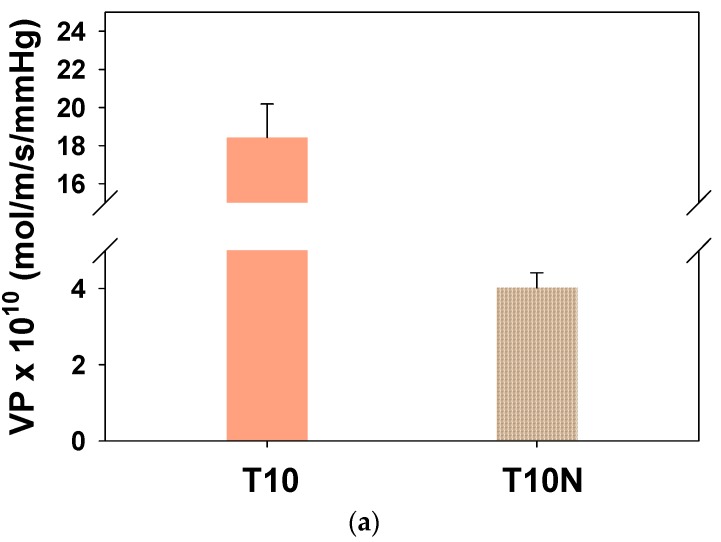

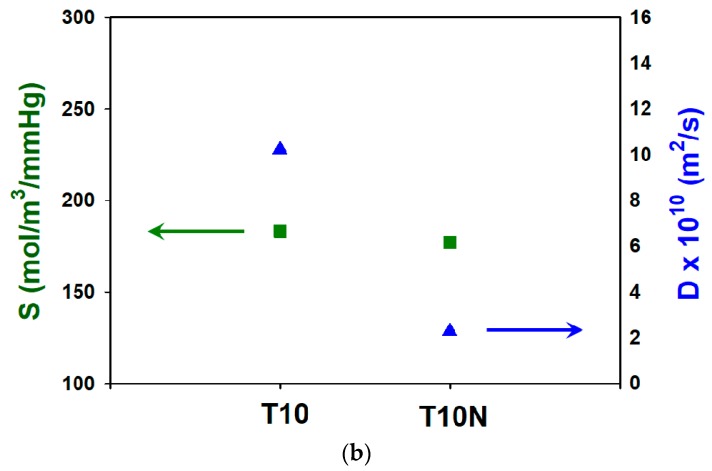
Dimethyl methylphophonate (DMMP) (**a**) permeabilities and (**b**) sorption and diffusion coefficients for T10 and T10N.

Considering safety concerns, simulants are generally used to replace chemical warfare agents. DMMP is a simulant of Sarin, which is one of the organophosphorus nerve agents, due to the comparable molecular size (0.57 nm for DMMP and 0.58 nm for Sarin) and water solubility [17,38]. Figure 11a shows the effect of the presence of nanofibers on DMMP penetration through PAMPS, and DMMP permeability of T10N is drastically lower than that of T10. In Figure 11b, it is seen that the sorption coefficient difference between T10 and T10N is not significant, while the diffusion coefficient is greatly decreased by the introduction of nylon nanofibers in PAMPS.

Generally, the composite membranes containing nanofillers exhibit a lower permeability due to the lower sorption caused by a reduced polymer matrix volume, as well as lower diffusion by increasing the tortuosity of pathways for permeants compared to those of pristine membranes [13]. For T10N, PAMPS is a permeable phase and the volume faction becomes lower than that of T10, which results in lower sorption coefficients and permeabilities. The lengthened pathways of water vapors caused by the presence of nanofibers also lead to a reduction in both water and DMMP permeabilities of T10N.

**Figure 12:**
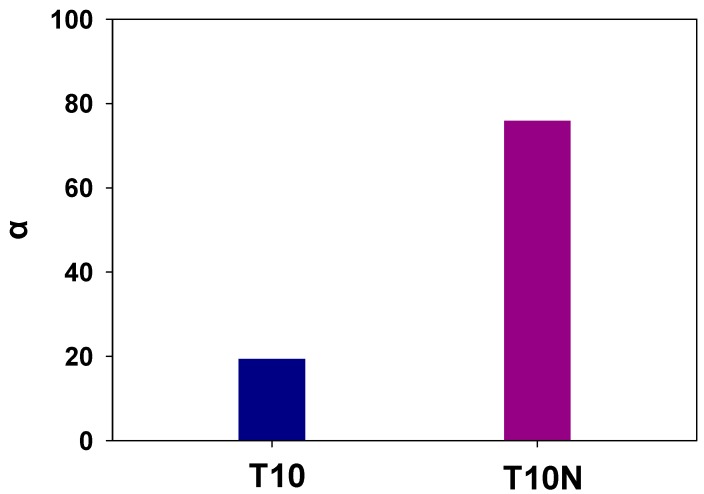
Selectivities of water over DMMP for T10 and T10N for T10 and T10N.

Selectivities of water over DMMP are calculated by dividing water permeabilities by DMMP permeabilities, and the effect of introducing nanofibers in PAMPS is shown in Figure 12. It shows that the selectivity of T10N is four times higher than that of T10. 

Even though its selectivity is lower than that of T10N, T10 still shows a high selectivity of 20. Sulfonic acid groups of PAMPS can dissociate into charged moieties in the presence of water, which makes them highly hydrophilic. Thus, the strong interaction between a membrane matrix (PAMPS) and a permeant (water) enables these to permeate water molecules preferentially and to prevent permeation of organic chemicals [39]. In addition, the diameter of DMMP (0.57 nm) is much larger than that of water molecules (0.25 nm), and thus, the size discrimination of permeant molecules causes a high selectivity of water over DMMP. In addition, the permeation of a larger molecule, DMMP, is more disturbed by the introduction of nanofillers compared to water permeation, resulting in a high selectivity of crosslinked PAMPS membranes containing nylon nanofibers. 

## 4. Conclusions

The water permeability of crosslinked PAMPS membranes using crosslinkers with different crosslinking lengths was studied. The sorption and diffusion were also observed to understand their permeation behavior. The results showed that PAMPS crosslinked with the larger crosslinkers exhibit a lower permeability caused by lower sorption despite higher diffusion. For chemical protection applications, electrospun nanofibers were introduced in the crosslinked PAMPS membranes to control permeability and selectivity. It was shown that both water and DMMP permeabilities are decreased by the presence of nanofibers. In addition, the reduction in DMMP permeability is more significant than that in water due to the high tortuosity of pathways for large permeants in the composite membranes, resulting in a high selectivity. The high selectivity of crosslinked PAMPS membranes containing nylon nanofibers, which allow for water transport while blocking the permeation of toxic and hazardous chemicals, indicates their potential as chemical protective membranes.

## Figures and Tables

**Table 1:** Mechanical properties of PAMPS membranes.

Sample	Young’s Modulus (MPa)	Tensile Strength (MPa)	Elongation at Break (%)
E10	0.84 ± 0.11	0.14 ± 0.04	76.1 ± 7.2
T10	0.80 ± 0.07	0.09 ± 0.02	60.1 ± 2.8
P10	0.77 ± 0.13	0.05 ± 0.02	41.0 ± 11.9
T10N	0.73 ± 0.07	0.06 ± 0.02	76.7 ± 18.6
